# How Bacteria Shift Gears

**DOI:** 10.1371/journal.pbio.1001061

**Published:** 2011-05-10

**Authors:** Robin Meadows

**Affiliations:** Freelance Science Writer, Fairfield, California, United States of America

**Figure pbio-1001061-g001:**
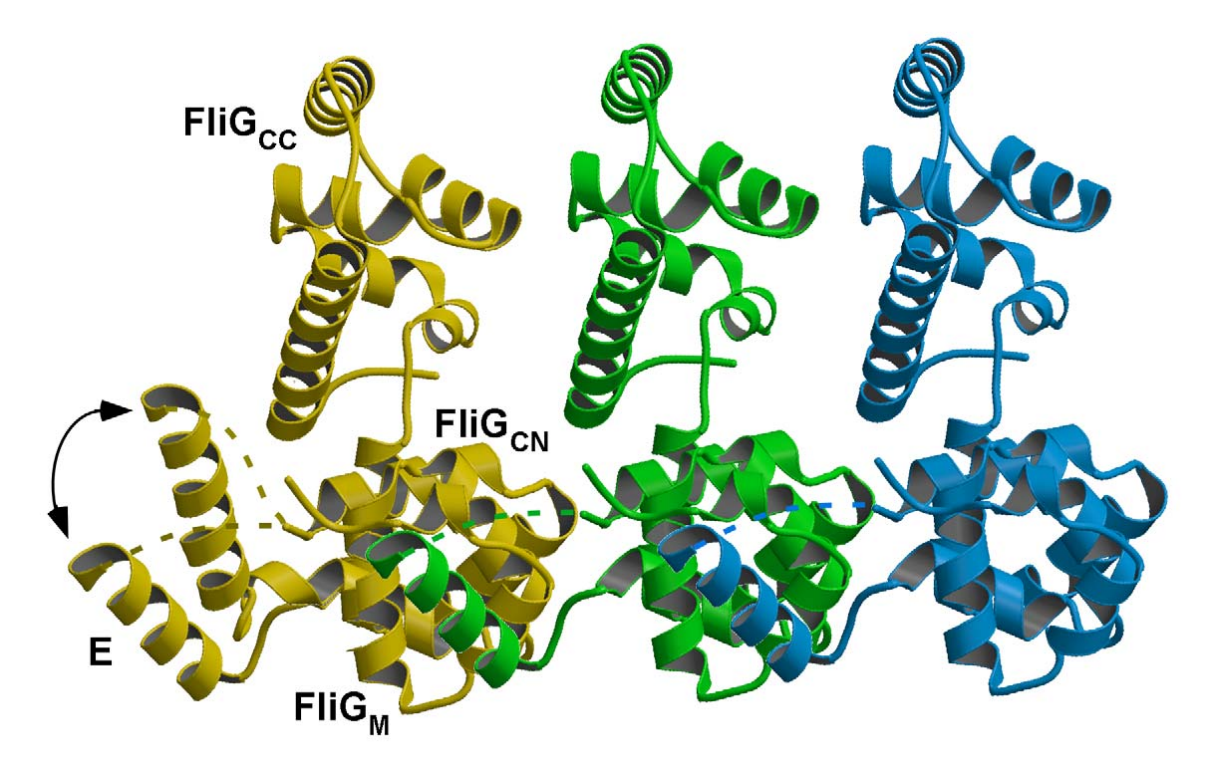
The motion of helix E caused by the conformational change of the hinge between FliG_M_ and helix E is key to the cooperative switching of flagellar motor rotation.

[Fig pbio-1001061-g001]Bacteria can change course almost instantaneously, zipping towards food or away from toxins. How do such simple organisms do something so complex? It's all in the flagella, a tail-like structure with rotating helical filaments. The flagella work in unison to propel the cell forward by rotating counterclockwise and thus bundling together. When the flagella reverse their rotation to clockwise, they disrupt the bundle and make the cell tumble in place. When the flagella shift back to counterclockwise again, the bacteria set off on a new course.

This description of bacterial locomotion is well known, but the mechanisms that allow the flagella to shift gears from counterclockwise to clockwise have proven difficult to identify. Now, in a new study in this issue of *PLoS Biology*, Katsumi Imada, Tohru Minamino and colleagues bring us closer to answering this fundamental question and propose a new model describing how flagella manage this switch.

Filaments in the flagella are powered by rotary motors that span the cell membrane. Things of beauty, these motors are tooled so precisely that they are nearly 100% efficient, and their direction is set by a rotor that can turn thousands of revolutions per minute. The rotor shifts from the forward-propelling counterclockwise to the tumble-inducing clockwise when chemical gradients tell bacteria they've gone astray, for example, away from food. This activates a cytoplasmic signaling protein that binds proteins in the rotor switch, changing the orientation of another switch protein called FliG and thereby reversing the rotor's spin to clockwise.

The details of the switch mechanism had been hypothesized but were as yet unproven. Previous X-ray crystallography studies of a FliG fragment had shown that two of its domains (FliG_M_ and FliG_C_) are connected by a helical linker called helix E, and the 3-D structure of a FliG protein predicted from its DNA sequence suggested that helix E might be flexible enough to make a good molecular switch. This suggestion was further supported by a 2010 report that compared the structure of a full-length FliG to the fragment: helix E was tightly packed in closed conformation in the full-length structure, but was in open conformation and dissociated from FliG_M_ in the fragment.

To find out if helix E is indeed the molecular switch that sets the direction of rotor spin, the researchers compared wild-type and mutant FliG fragments containing the two domains linked by helix E. The wild-type motors were set to spin counterclockwise by experimental conditions, and the mutant had a type of amino acid deletion that sets the rotor spin to clockwise. As expected, X-ray crystallography revealed that the wild-type (counterclockwise) and mutant (clockwise) FliG fragments had different helix E conformations.

The difference was in the hinge between helix E and FliG_M_, reorienting the former and exposing part of the latter in the mutant fragments, suggesting that this hinge may be the molecular switch that shifts FliG's orientation between counterclockwise and clockwise states. This conclusion was strengthened by the finding that while the FliG proteins studied came from several bacteria species and varied considerably, they shared a conserved FliG_M_-FliG_C_ element.

Based on their discovery, the researchers propose a new model for rotational switching in bacterial flagella. The rotor base has a ring of FliG subunits that switch cooperatively between counterclockwise and clockwise states. The model holds that besides affecting the orientation of its own subunit, the hinge between helix E and FliG_M_ also affects the orientation of the neighboring FliG subunit. Thus, conformational change of this molecular switch rapidly spreads from subunit to subunit, thus propagating it all around the ring.

This work provides the most direct evidence yet that helix E is the molecular switch underlying the flagellar motor's gear shift from counterclockwise to clockwise, as well as the most complete model of the cooperative flagellar switch. Besides advancing our understanding of the flagellar motor, which is a marvel of nature, this study could help lay the groundwork for developing drugs that target key motor proteins and so immobilize harmful bacteria.

**Minamino T, Imada K, Kinoshita M, Nakamura S, Morimoto YV, et al. (2011) Structural Insight into the Rotational Switching Mechanism of the Bacterial Flagellar Motor. doi:10.1371/journal.pbio.1000616**

